# House Dust Mite Aeroallergen Suppresses Leukocyte Phagocytosis and Netosis Initiated by Pneumococcal Lung Infection

**DOI:** 10.3389/fphar.2022.835848

**Published:** 2022-02-22

**Authors:** Angelica Papanicolaou, Hao Wang, Jonathan McQualter, Christian Aloe, Stavros Selemidis, Catherine Satzke, Ross Vlahos, Steven Bozinovski

**Affiliations:** ^1^ School of Health and Biomedical Sciences, RMIT University, Bundoora, VIC, Australia; ^2^ Translational Microbiology Group, Murdoch Children’s Research Institute, Parkville, VIC, Australia; ^3^ Department of Paediatrics, The University of Melbourne, Parkville, VIC, Australia; ^4^ Department of Microbiology and Immunology, The Peter Doherty Institute for Infection and Immunity, The University of Melbourne, Melbourne, VIC, Australia

**Keywords:** asthma, house dust mite, *Streptococcus pneumoniae*, phagocytosis, NEtosis

## Abstract

Asthmatics are highly susceptible to developing lower respiratory tract infections caused by *Streptococcus pneumoniae* (SPN, the pneumococcus). It has recently emerged that underlying allergic airway disease creates a lung microenvironment that is defective in controlling pneumococcal lung infections. In the present study, we examined how house dust mite (HDM) aeroallergen exposure altered immunity to acute pneumococcal lung infection. Alveolar macrophage (AM) isolated from HDM-exposed mice expressed alternatively activated macrophage (AAM) markers including YM1, FIZZ1, IL-10, and ARG-1. *In vivo*, prior HDM exposure resulted in accumulation of AAMs in the lungs and 2-log higher bacterial titres in the bronchoalveolar (BAL) fluid of SPN-infected mice (Day 2). Acute pneumococcal infection further increased the expression of IL-10 and ARG1 in the lungs of HDM-exposed mice. Moreover, prior HDM exposure attenuated neutrophil extracellular traps (NETs) formation in the lungs and dsDNA levels in the BAL fluid of SPN-infected mice. In addition, HDM-SPN infected animals had significantly increased BAL fluid cellularity driven by an influx of macrophages/monocytes, neutrophils, and eosinophils. Increased lung inflammation and mucus production was also evident in HDM-sensitised mice following acute pneumococcal infection, which was associated with exacerbated airway hyperresponsiveness. Of note, PCV13 vaccination modestly reduced pneumococcal titres in the BAL fluid of HDM-exposed animals and did not prevent BAL inflammation. Our findings provide new insights on the relationship between pneumococcal lung infections and allergic airways disease, where defective AM phagocytosis and NETosis are implicated in increased susceptibility to pneumococcal infection.

## Introduction

Asthma is a chronic airway inflammatory disease characterised by pathological airway remodelling that leads to variable and reversible airflow obstruction. A major consequence of chronic asthma and its related immunopathology is that asthmatics can be particularly vulnerable to respiratory infections, which can trigger potentially life-threatening acute exacerbations. Asthmatics have a heightened risk of developing pneumonia caused by *Streptococcus pneumoniae* (SPN, the pneumococcus) (Juhn et al., 2008; Castro-Rodriguez et al., 2020), which is a leading cause of community acquired pneumonia (CAP). The risk of hospitalisation by pneumonia is 2–4 times higher in asthmatics, where severe asthmatics are at even greater risk (Ekbom et al., 2019). This could be due to increased pneumococcal oropharyngeal carriage in both adults and children with asthma (Jounio et al., 2010; Esposito et al., 2016), as the pneumococcus usually infects the lungs by escaping from the upper respiratory tract (Bogaert et al., 2004). The risk of developing asthma is also significantly increased in children that are colonised with pathogenic bacteria including SPN in the upper airways as neonates (Bisgaard et al., 2007). Hence, asthmatics are regarded as a high-risk group where pneumococcal disease can be more severe. Protection of such “high-risk” asthmatics through vaccination continues to be an important priority, although it is unclear as to whether the efficacy of SPN vaccines is compromised in asthmatics (Sheikh et al., 2002).

Whilst SPN vaccines are central to reducing the risk of acquiring SPN and developing pneumonia, they may offer limited protection once SPN infects the lungs of asthmatics where the relationship between asthma and S. *pneumoniae* is complex. Alveolar macrophages (AMs) are the first cellular defence to bacterial pathogens entering the lungs (Franke-Ullmann et al., 1996) where they coordinate operational phagocytosis, which is essential for the removal of pathogenic microorganisms. AMs isolated from adults with severe asthma, phagocytose bacterial pathogens such as *H. influenzae* and *S. aureus* at a reduced capacity (Liang et al., 2014). A similar phenomenon is also observed in children with severe asthma (Fitzpatrick et al., 2008). Type-2 immune cytokines such as IL-4 and IL-13 can also drive the emergence of alternatively activated macrophage (AAM) phenotypes, which express a distinct set of genes that may compromise pathogen control (Varin et al., 2010; Abdelaziz et al., 2020). In addition to AM-mediated pathogen control, neutrophils play a pivotal role in clearing remaining pneumococci within the alveolar spaces. Neutrophil-mediated clearance of SPN involves both the killing of phagocytosed bacteria with stored serine proteases like neutrophil elastase and cathepsin G (Standish and Weiser, 2009), and the formation of neutrophil extracellular traps (NETs) (Mori et al., 2012); a process called NETosis. NETs are found in bronchial biopsies from patients with allergic asthma (Dworski et al., 2011), and their *ex vivo* formation is inversely correlated to lung function (Pham et al., 2017).

How phagocytic cells deal with SPN infections within the allergic airways is still being defined at a cellular and molecular level and there is conflicting data. For example, reduced TLR2 expression in lung phagocytic cells of house dust mite (HDM)-sensitised mice resulted in a failure to effectively clear SPN due to reduced neutrophil migration into the lungs (Habibzay et al., 2012). Contrastingly, in an OVA-model of allergic disease, there was a similar reduction in neutrophil migration in response to acute SPN infection; however, this was associated with an improvement in bacterial clearance due to expansion of a distinct SiglecF^low^ macrophage population (Sanfilippo et al., 2015). In this study, we examined whether acute pneumococcal lung infection altered the phenotype and function of AMs following HDM exposure. We also evaluated the formation of NETs following SPN infection of HDM-sensitised mice and assessed whether the pneumococcal conjugate vaccine (PCV13) restores dysfunctional bacterial clearance in HDM-sensitised mice.

## Methods and Materials

### Mice and Experimental Asthma Exacerbation Model

Specific pathogen-free 6-week-old female BALB/C mice were purchased from the Animal Resources Centre (Western Australia). All mice were housed under normal 12-h day/night cycle at 22°C with free access to food and water. All animal experiments were approved by the Animals Ethics Committee of RMIT University (AEC #1805) and conducted in compliance with the National Health and Medical Research Council of Australia guidelines for experimental animal use and care. Mice were lightly anaesthetised in an induction chamber filled with 4% isoflurane in oxygen and 25 μg of HDM ([D. Pteronyssinus], Stellergenes Greer, United States) in 35 μL of saline was intranasally administered into the nostrils. In total, mice were exposed to HDM aeroallergen three times per week for 3 weeks. Vehicle exposed mice were treated in the same manner receiving 35 μL of saline alone. Twenty-four hours after the last HDM challenge, mice were inoculated with 10^6^ CFU *S. pneumoniae* (strain EF3030, serotype 19F), in 35 ml saline delivered by intranasal delivery under light anaesthesia. A separate cohort of mice were vaccinated with the pneumococcal conjugate vaccine (Prevnar 13 [PCV13], Pfizer, United States), where the vaccine was diluted 1:10 with saline prior to administration. Mice received PCV13 via intraperitoneal injection (100 μL per mouse) 1 week prior to initiation of the HDM protocol and a second booster shot was delivered 2 weeks later, as previously described (Ohori et al., 2020).

### Isolation of Alveolar Macrophages by Flow Cytometry and Phagocytosis Assay

Mice were killed by an overdose of sodium pentobarbital (240 mg/kg, Virbac, Australia) and lung lobes from saline or HDM challenged mice were dissected and finely minced in a petri dish. Lung cells were dissociated in 4 ml of Liberase^™^ (Sigma-Aldrich, United States) at 37°C. Following removal of residual tissue debris and erythrocytes, single cell suspensions were stained with an antibody cocktail including Anti-CD45-FITC, Anti-F4/80-APC, Anti-CD11b-eFluor450, and anti-CD11c-PE-Cy7 (Thermo Fisher Scientific, United States). Alveolar macrophages were sorted using the FACSAria^™^ Fusion (BD Biosciences, United States) under a strict gating strategy. Briefly, dead cells, cellular debris, and cell doublets were excluded using PI viability dye, side scatter (SSC), and forward scatter (FCS) gates. From this population of live singlets, haemopoietic cells were selected based on high CD45^+^ expression. All F4/80- cells were then excluded from the analysis, and alveolar macrophages were differentiated based upon CD11c^high^ and CD11b^low^ expression. Freshly isolated alveolar macrophages were used for mRNA extraction.

### Lung Function, and Assessment of BAL and Lung Inflammation

Mice were anesthetised by ketamine (125 mg/kg) and xylazine (25 mg/kg), after which tracheotomy was performed by inserting an 18G canular into the trachea. Airway reactivity in response to increased doses of methacholine (MCh) was measured *in vivo* with the Flexivent FX1 (SCIREQ^®^ Montreal, QC, Canada) as previously described (Wang et al., 2019). Mice were subsequently killed by an overdose of sodium pentobarbital (240 mg/kg, Virbac, Australia) and lungs were then lavaged using the same cannula with a total volume of 1.3 ml PBS to retrieve the bronchoalveolar (BAL) fluid, as previously described (Wang et al., 2017; Wang et al., 2019; Wang et al., 2021). The total number of viable BAL cells was determined by acridine orange/ethidium bromide viability staining and a hemocytometer chamber. BAL cytospins were prepared by centrifugation using the Cytopsin 3 (Thermo Shandon, United States) at 400 x g for 10 min. Cytospins were air dried, stained using the Kwik-Diff Kit^®^stain (Thermo Fischer Scientific, United States) procedure and cover slipped, as per the manufacturer’s instructions. Differential cell counts were determined based on standard morphological features observed under microscopy with a minimum of 300 cells per slide enumerated. Representative images of macrophages were taken using brightfield microscopy (BX60 microscope, Olympus, Japan).

Following BAL, the left lung lobe was removed and fixed in 10% neutral buffered formalin for histological processing. In order to assess lung inflammation, H&E-stained slides were blindly scored by two assessors for peribronchiolitis and alveolitis, where lung lobe sections were scored based on a scale of 1–5: 0, healthy lungs i.e. no inflammation; 1, very mild; 2, mild; 3, moderate; 4, severe; 5, extremely severe changes, as previously described (Wang et al., 2021). Alcian blue-periodic acid-Schiff (AB-PAS)-stained slides were also analysed to evaluate presence of goblet cell hyperplasia. Slides were captured using a whole slide scanner and analysed using the Olympus CellSens software (V1.18, Olympus, Japan). The mucus positive area fraction (%) within the airways was calculated for four small airways (100–400 μm in diameter) per section using a standardised hue, saturation and value threshold consistent across all samples.

### Bacterial Enumeration in Nasal Tissue and Lungs

Nasal tissue was dissected and placed in 1 ml of Dulbecco’s Modified Eagle Medium ([DMEM], Gibco^™^, Thermo Fischer Scientific, United States) prior to homogenisation using the T18 digital ULTRA-TURRAX^®^ (IKA, Germany). Pneumococcal loads were determined by viable count using BAL fluid and homogenised nasal tissue, which was serially diluted 10-fold in PBS. 15 μL of each dilution was pipetted onto horse blood agar (HBA) + 5% gentamycin (5 μg/ml) plates (Media Preparation Unit, The University of Melbourne, Australia) in triplicate. Following overnight incubation, the highest dilution with countable colonies was enumerated and the number of viable pneumococci was expressed as CFU/mL.

### Immunohistochemistry for NETs and dsDNA Determination

Unstained lung tissue slides were rehydrated and incubated in antigen retrieval buffer (Tris-EDTA Buffer, 10 mM Tris, 1 mM EDTA, 0.05% Tween 20, pH 9.0). Slides were permeabilised in PBS 0.5% Triton X-100 and blocked (5% horse serum + 5% BSA + 300 mM Glycine in PBS-Tween 0.05%). Slides were then incubated with goat anti-mouse MPO (R&D Systems, United States) and rabbit anti-mouse citrullinated histone H3 (Abcam, United Kingdom) for 2 h, washed and incubated with the secondary antibodies Alexa Fluor 568 donkey anti-rabbit IgG and Alex Fluor 488 donkey anti-goat IgG. Slides were washed three times in PBS-Tween and coverslips were mounted using Fluoroshield^™^ with DAPI histology mounting medium (Sigma-Aldrich, United States). The mean overlapping histone-MPO area fraction (%), depicted by the yellow staining regions, was determined using a whole slide scanner and the Olympus CellSens software (V1.18, Olympus, Japan). The concentration of dsDNA in the BAL fluid was measured using the Quant-iT^™^ PicoGreen^™^ Assay Kit (Thermo Fischer Scientific, United States) as per the manufacturer’s instructions.

### RTqPCR Analysis of Isolated Alveolar Macrophages and Lung Tissue

Frozen lung tissue was ground to a fine powder using a mortar and pestle. RNA from isolated alveolar macrophages or lung tissue were purified using the RNeasy mini kit as per manufacturers instruction (Qiagen, GMH Germany). The NanoDrop200 (Thermo Fischer Scientific, United States) was used to determine the concentration and purity of extracted RNA and the High-Capacity RNA to cDNA kit (Thermo Fischer Scientific, United States) was used to generate complementary DNA (cDNA). RT-qPCR was performed using the QuantStudio 7 system (Thermo Fischer Scientific, United States), TaqMan primers and the Taqman Fast Advanced Master Mix, as per manufacturer’s instructions. The relative expression of each gene was normalised against the housekeeping gene glyceraldehyde phosphate dehydrogenase (GAPDH) and determined using the ΔΔCt value method, as previously described (Wang et al., 2018; Wang et al., 2019).

## Results

### HDM Aeroallergen Compromised Clearance of SPN in the Lungs

**FIGURE 1:**
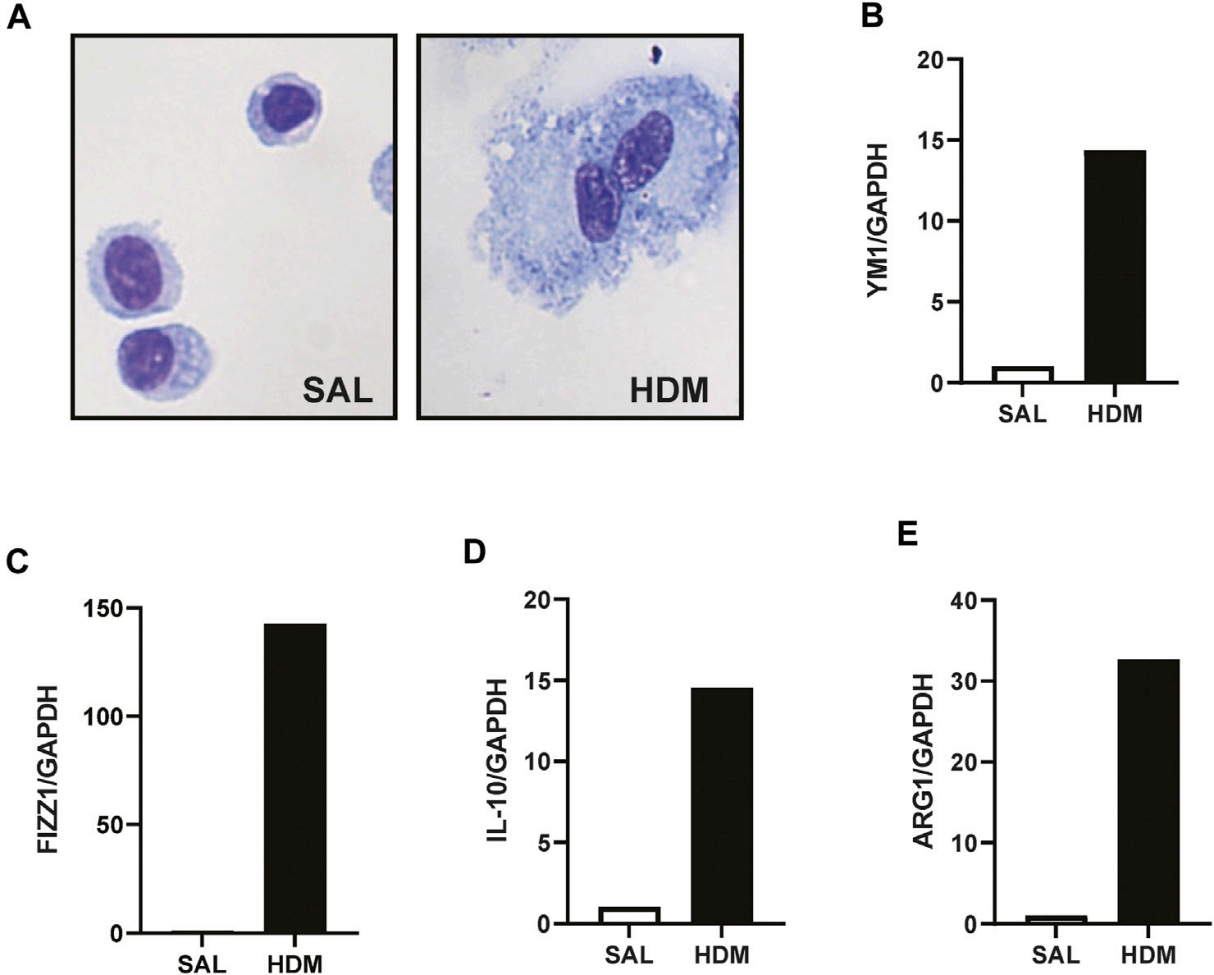
HDM aeroallergen alters the phenotype of lung macrophages. **(A)** Alveolar macrophages were isolated by FACS sorting from the lungs of mice exposed to saline (SAL) or house dust mite (HDM) aeroallergen over 3 weeks and images of representative cytospins were taken. **(B–E)** The expression of alternative activation markers including YM-1, FIZZ1, IL-10, and ARG1 were analysed by RTqPCR using Taqman primers. Data was generated using pooled alveolar macrophages from *n* = 5–10 mice per treatment group. Data are presented as mean ± SEM.

**FIGURE 2:**
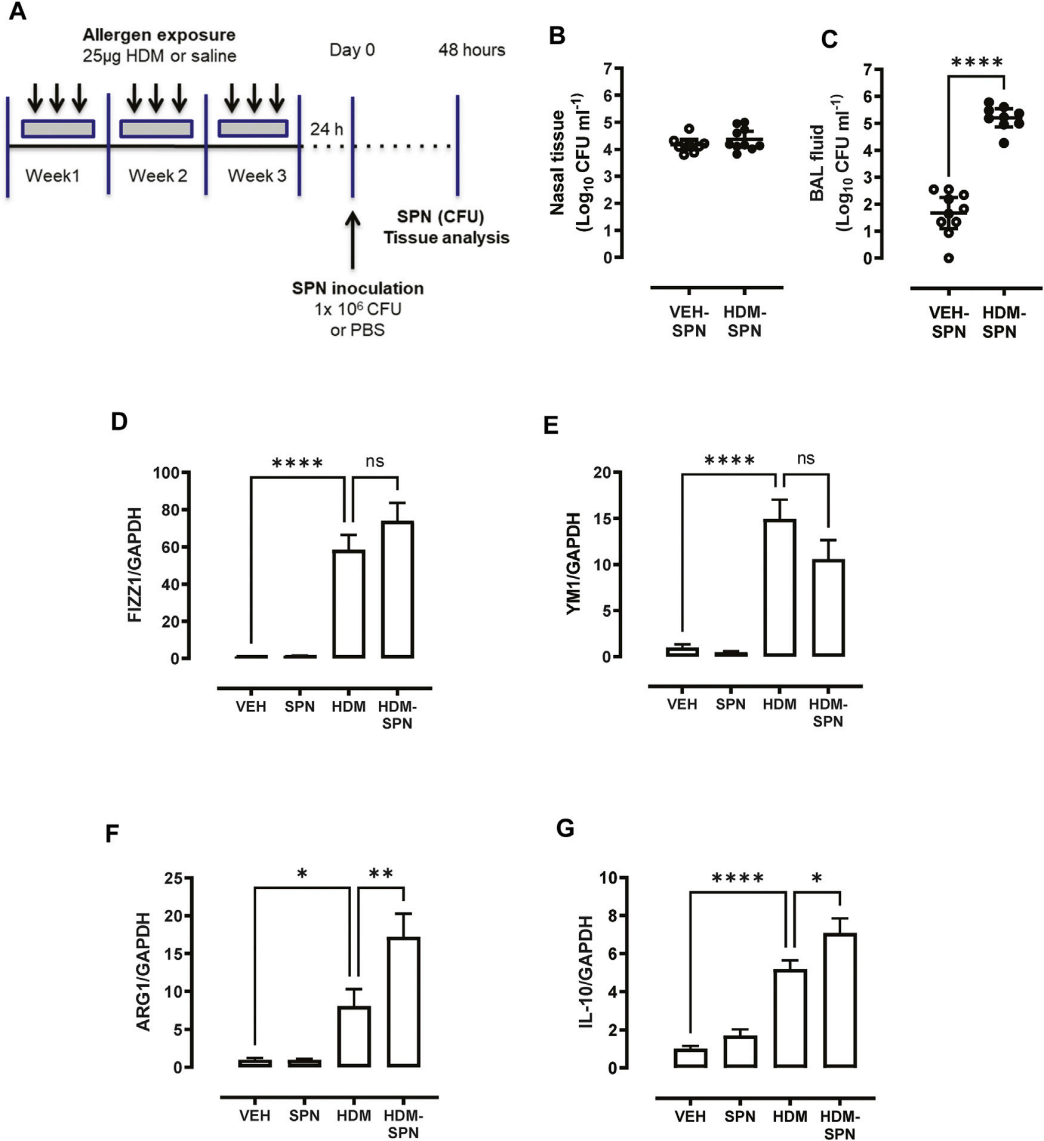
HDM suppresses bacterial clearance and increases IL10/ARG1 expression in SPN-infected mice. **(A)** Mice were sensitized to HDM over three 3 weeks and subsequently infected with SPN *via* intranasal inoculation, and outcomes were assessed 2 days later. Pneumococcal load in the **(B)** nasal tissue and **(C)** BAL fluid was determined. **(D–G)** Expression of the alternative activation markers FIZZ1, YM1, ARG1, and IL-10 in lung tissue was analysed by RTqPCR using Taqman primers. *n* = 7–10 mice per group. RT-qPCR data are presented as mean ± SEM and statistical comparisons were determined using one-way ANOVA at *p < 0.05, **p < 0.01, ****p < 0.0001, followed by Tukey’s *post hoc* test. SPN titres are presented as the geometric mean ± 95% confidence interval (CI) and statistical comparisons were determined using the Mann-Whitney U test at ****p < 0.0001.

AMs were isolated using a cell sorting procedure from mice exposed to HDM aeroallergen or saline for 3 weeks. Freshly isolated AMs from control (saline) mice maintained a stereotypical AM morphology, whereas AMs isolated from HDM-exposed mice displayed an enlarged and highly vacuolated morphology (Figure 1A). The change in morphology was associated with a marked increase in the expression of the two alternatively activated macrophage (AAM) markers, YM1, and FIZZ1 (Figures 1B, C). In addition, IL-10 and ARG1 expression was markedly increased in HDM-exposed AMs (Figures 1D, E). To determine whether prior HDM exposure impacted pneumococcal clearance *in vivo*, HDM-sensitised mice were acutely infected with SPN and bacterial titres were evaluated 48 h following SPN inoculation as summarised in Figure 2A. Pneumococcal loads remained consistent between HDM and VEH-exposed mice in the nasal tissue (Figure 2B); however, SPN titres in the BAL fluid were over 3-log higher in HDM-exposed mice compared to VEH-exposed mice (Figure 2C). We next assessed whether an AAM population had emerged *in vivo* as seen in the isolated AM population, where the common AAM markers (FIZZ1, YM1, ARG1, and IL-10) were measured in lung *via* RT-qPCR. HDM sensitisation increased the expression of all four AAM markers and of note, combined HDM exposure and acute SPN infection (HDM-SPN) lead to a further increase in the relative expression of IL-10 and ARG1 compared to HDM alone-exposed mice (Figures 2D–G).

**FIGURE 3:**
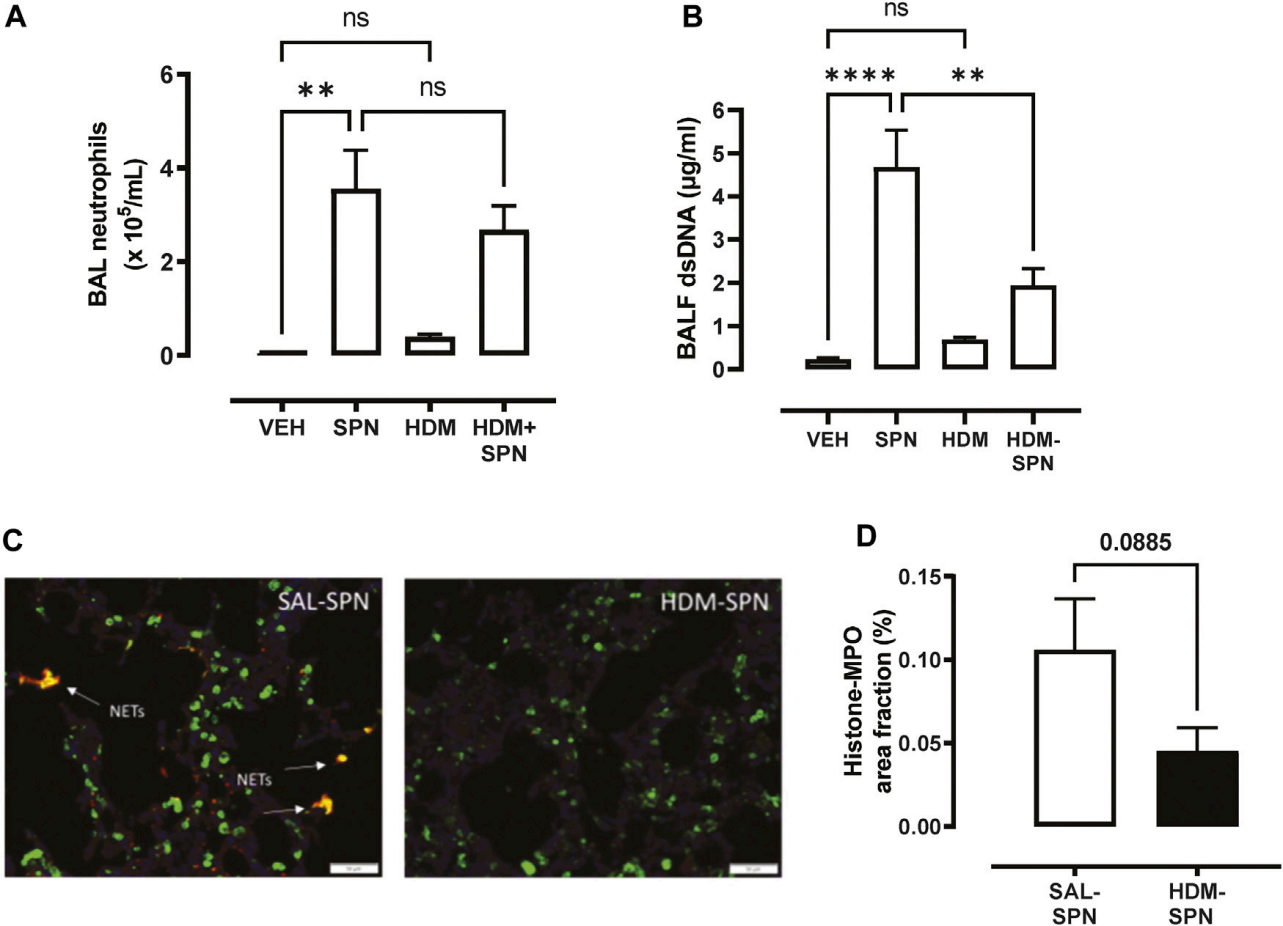
Netosis markers are reduced in HDM-exposed mice infected with SPN. **(A)** BAL neutrophil numbers were enumerated on BAL cytospots. **(B)** The levels of dsDNA were quantified in the BAL fluid. **(C)** NET staining was performed on lung sections and identified yellow regions of H3 and MPO co-staining that is representative of NET formation. **(D)** The area of co-staining was quantified on a slide scanner using cell sense software in SAL and HDM treated mice infected with SPN. *n* = 6–10 mice per group. Data are presented as mean ± SEM and statistical comparisons were determined using one-way ANOVA for more than two groups, or Students *t-test* for two groups at **p < 0.01, ****p < 0.001, followed by Tukey’s *post hoc* test.

Since pneumococcal lung infection promotes the migration of neutrophils into the airspaces to assist with pathogen removal, BAL neutrophils were analysed in our model. There was a significant increase in the number of BAL neutrophils in response to acute pneumococcal infection and the numbers were not significantly altered by prior HDM exposure (Figure 3A). The levels of dsDNA in the BAL fluid were also measured as a marker for the release of NETs, where dsDNA levels were significantly increased in mice infected with SPN (Figure 3B). Of importance, there was a marked reduction in dsDNA levels in the BAL fluid in mice exposed to HDM prior to SPN infection relative to SPN-infected mice. Complementing these findings, lung sections were co-stained for citrullinated histone H3 (red) and MPO (green), two key components of NETs. Overlapping yellow regions were used to define regions of NETs, which were observed in the lungs of SPN-infected mice, as presented in Figure 3C. The quantification of co-stained regions displayed a mean area fraction of 0.11% in SPN-infected mice, compared to just 0.045% in SPN-infected animals previously exposed to HDM, although this difference fell short of significance (Figure 3D).

### Increased Pneumococcal Load Worsened Inflammation and Airway Hyper-Responsiveness

**FIGURE 4:**
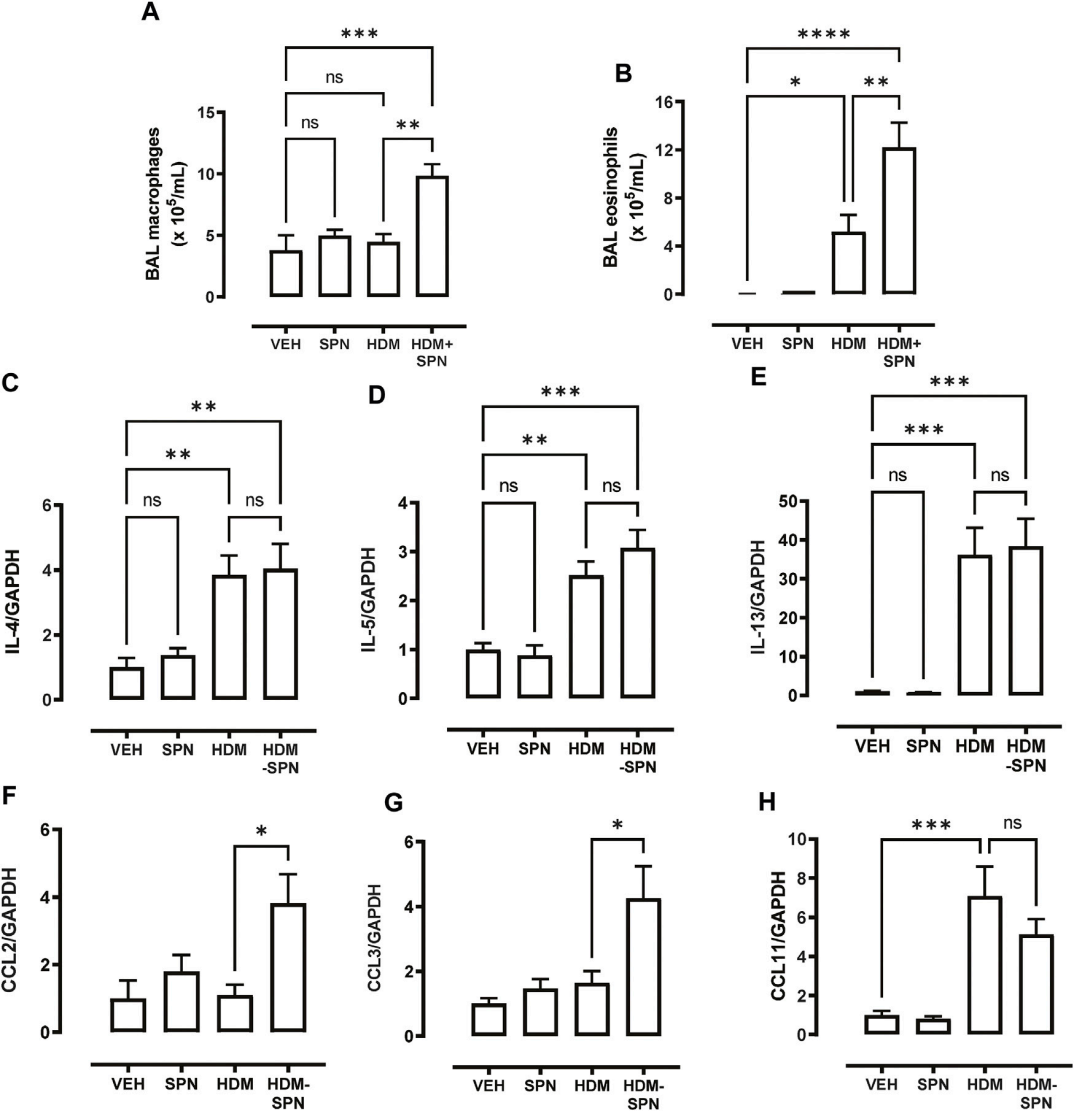
CCL2 and CCL3 are associated with increased BAL macrophages and eosinophils. **(A)** BAL macrophage and **(B)** BAL eosinophil numbers were enumerated on BAL cytospots. **(C–E)** Expression of the TH2 markers including IL4, IL5, and IL13 were analysed by RTqPCR using Taqman primers. **(F–H)** Expression of CCL chemokines including CCL2, CCL3, and CCL11 were analysed by RTqPCR using Taqman primers. *n* = 6–10 mice per group. Data are presented as mean ± SEM and statistical comparisons were determined using one-way ANOVA at *p < 0.05, **p < 0.01, ***p < 0.005, followed by Tukey’s *post hoc* test.

In addition to the influx of neutrophils, differential cell counting revealed that acute pneumococcal infection in HDM-exposed mice significantly increased the total number of BAL macrophages, whereas HDM or SPN alone did not alter BAL macrophage numbers (Figure 4A). In addition, eosinophilic inflammation induced by HDM exposure was further increased by concurrent acute pneumococcal infection in HDM-exposed mice (Figure 4B). To interrogate the molecular mechanisms that are driving this distinct BAL inflammatory profile in HDM-SPN mice, a panel of immunoregulatory genes were measured in the lungs by RTqPCR. As expected, the classic type-2 cytokines IL4, IL5, and IL-13 were increased in the lungs of HDM-exposed mice (Figures 4C–E), and pneumococcal infection did not alter their respective levels. In contrast, the expression of the CCL2 (MCP1) and CCL3 (MIP1a) chemokines were not increased by HDM or SPN alone; however the combination of HDM and SPN significantly increased their levels (Figures 4F, G). The expression of CCL11 (eotaxin-1) was increased in HDM-exposed mice and levels were not further altered with SPN infection (Figure 4H).

**FIGURE 5:**
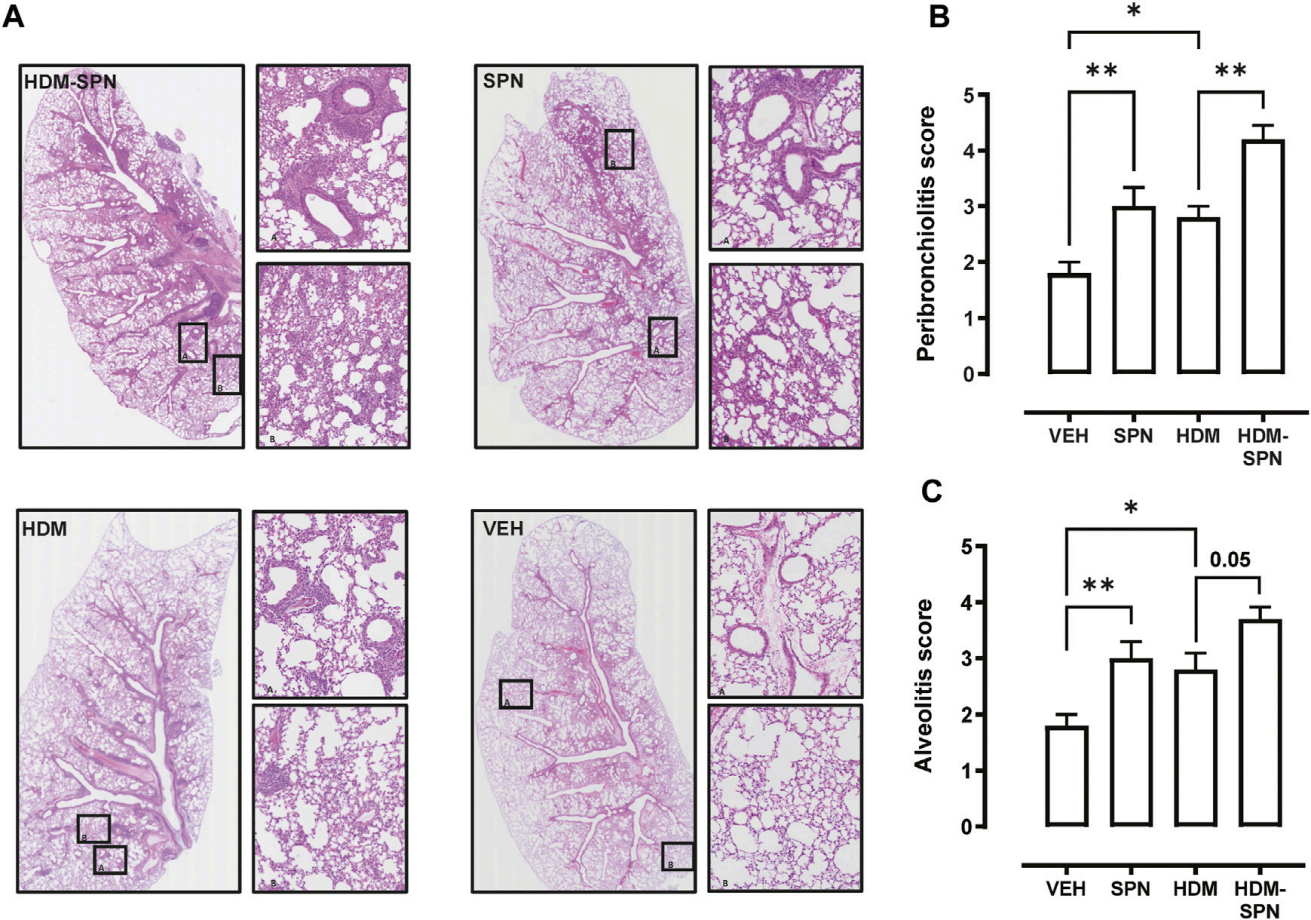
Lung inflammation and injury is increased in HDM-SPN infected mice. **(A)** Lungs were processed for histology and slides were stained with H&E, and whole lung lobes were captured using the VS120 slide scanner. The level of **(B)** peribronchial inflammation and **(C)** alveolitis was blindly scored by two observers. *n* = 7–10 mice per group. Data are presented as mean ± SEM and statistical comparisons were determined using one-way ANOVA at *p < 0.05, **p < 0.01, followed by Tukey’s *post hoc* test.

**FIGURE 6:**
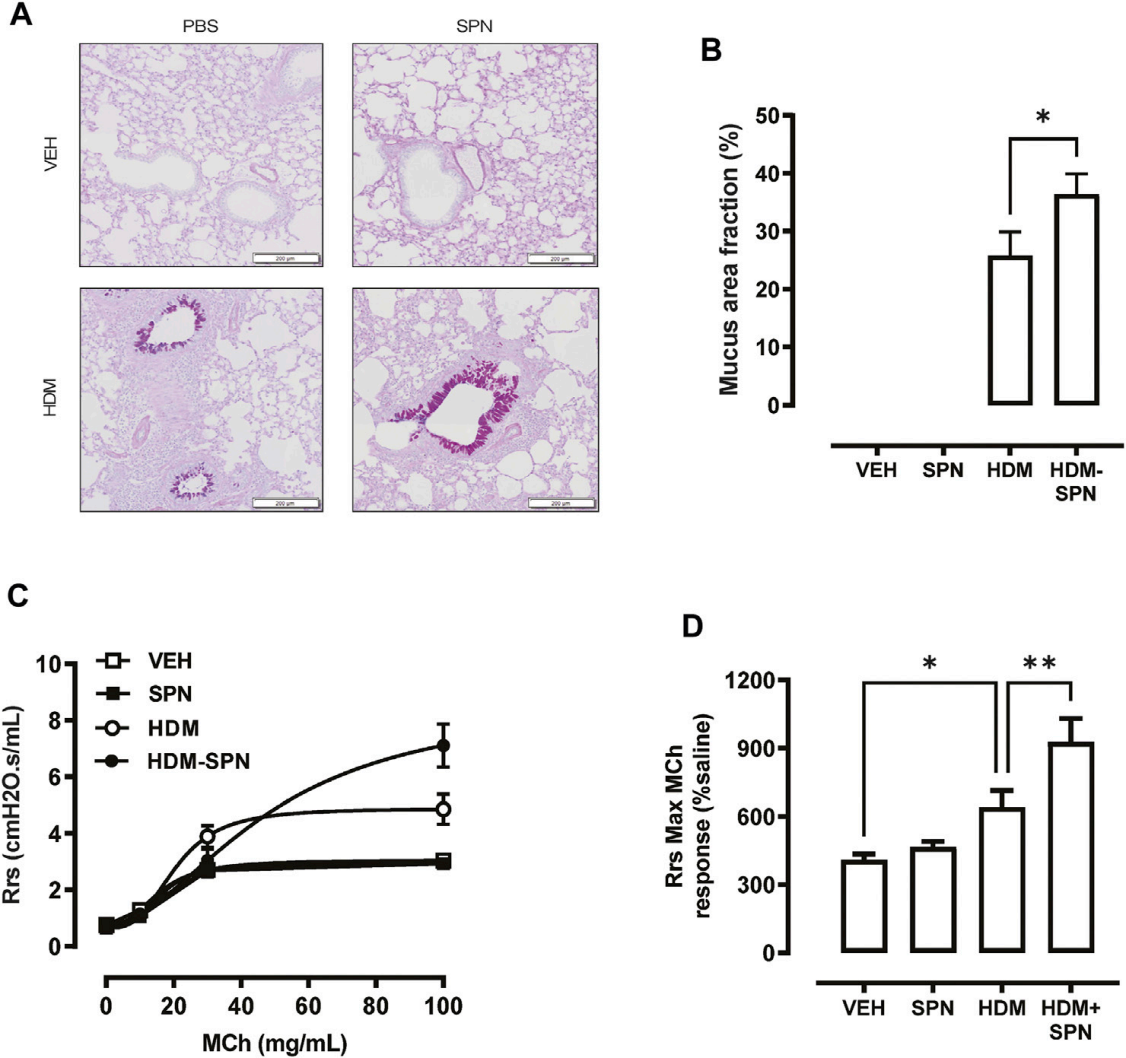
Mice sensitised with HDM and infected with SPN display increased airways hyper-reactivity. **(A)** Lungs were processed for histology and slides were stained with AB-PAS, and whole lung lobes were captured using the VS120 slide scanner. **(B)** The area of mucous staining within the airways was blindly evaluated using Cell Sense software. Lung function in response to increasing concentrations of methacholine was performed, where **(C)** total resistance (Rrs) was presented as a dose response and **(D)** maximal Rrs responses at 100 mg/ml MCh were compared across the different groups. *n* = 8–10 mice per group. Data are presented as mean ± SEM and statistical comparisons were determined using one-way ANOVA at *p < 0.05, **p < 0.01, followed by Tukey’s *post hoc* test.

We next investigated whether there were any noticeable histological changes to the airways and parenchyma. To determine the level of peribronchiolitis and alveolitis, whole-lung sections stained with H&E (Figure 5A) were blindly scored. SPN infection or HDM exposure alone resulted in increased peribronchiolar (Figure 5B) and alveolar inflammation (Figure 5C). The combined HDM-SPN exposure specifically led to a significant increase in peribronchiolitis when compared to the HDM alone group. Mucus production around the small airways (<200 μm) was also investigated via AB-PAS staining of the whole lung, where mucus positive cells were rarely observed within the airways of VEH or SPN treated mice (Figure 6A). HDM exposure resulted in a mean mucus staining airway wall area fraction of 25.77% that significantly increased to 36.35% with combined acute SPN infection (Figure 6B). Lung function parameters were also measured, and dose response analysis revealed that at the maximum nebulised dose of methacholine (100 mg/ml), total resistance (Rrs) peaked in HDM-SPN animals (Figure 6C). Comparison of maximal responses revealed that SPN did not significantly increase Rrs, whereas HDM alone increased Rrs, and the combination of HDM and SPN caused a further significant rise in Rrs (Figure 6D).

**FIGURE 7:**
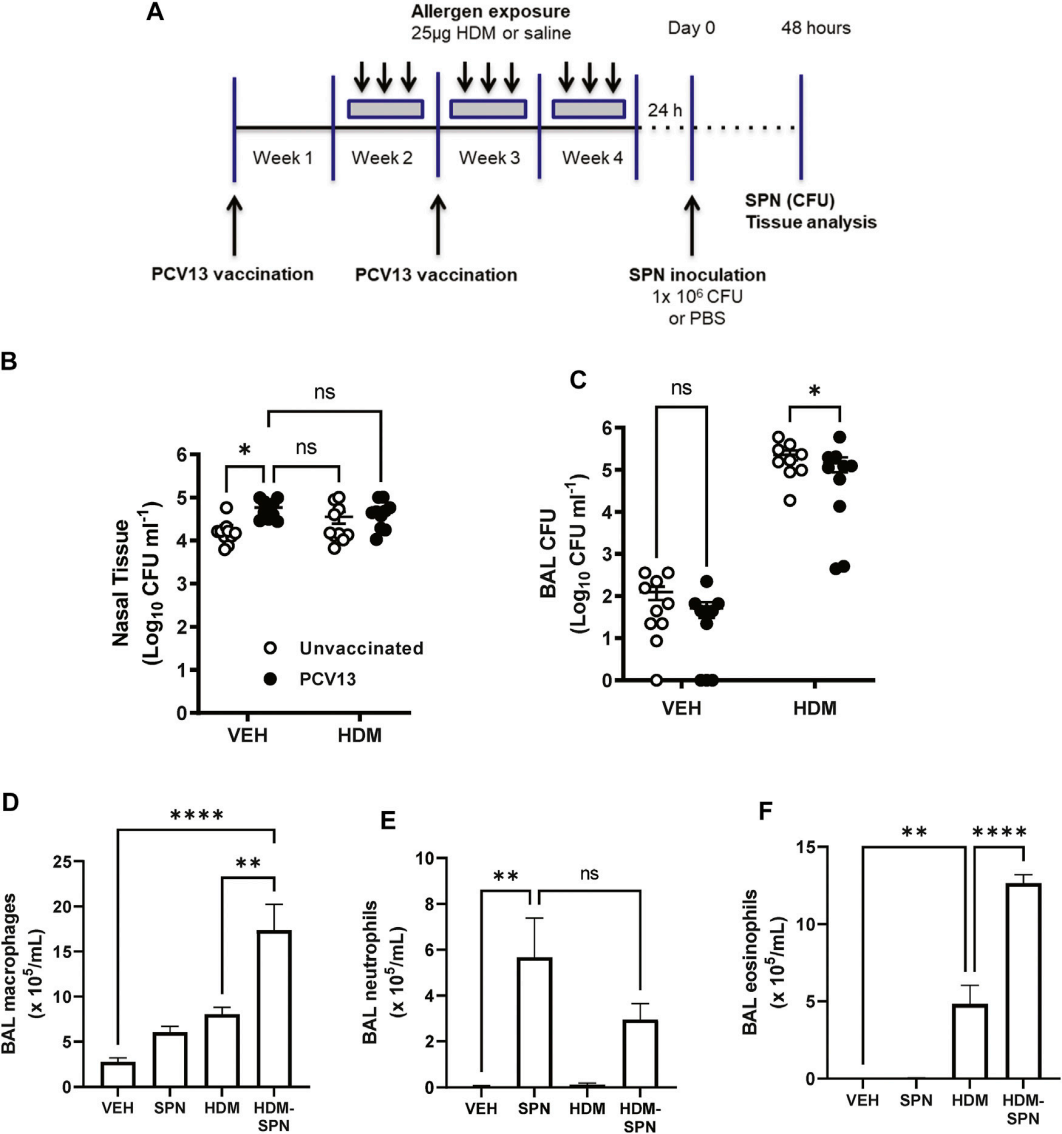
SPN lung clearance is improved in PCV13-vaccinated and HDM-exposed mice. **(A)** Mice were vaccinated with PCV13 at weeks 1 and 2, sensitized to HDM over three 3 weeks and subsequently infected with SPN *via* intranasal inoculation. Outcomes were assessed 2 days later. Pneumococcal load in the **(B)** nasal tissue and **(C)** BAL fluid was determined. **(D)** BAL macrophage, **(E)** BAL neutrophils, and **(F)** BAL eosinophil numbers were enumerated on BAL cytospots. *n* = 4-5 mice per group. SPN titres are presented as the geometric mean ± 95% confidence interval (CI) and statistical comparisons were determined using a mixed-effects model with Sidak’s *post hoc test* at *p < 0.05. BAL inflammatory cell numbers are presented as mean ± SEM and statistical comparisons were determined using one-way ANOVA at **p < 0.01, ****p < 0.0001, followed by Tukey’s *post hoc* test.

We next determined whether prior HDM exposure impacted pneumococcal clearance in PCV13 vaccinated mice by evaluating bacterial titres during the acute phase of infection as summarised in Figure 7A. Pneumococcal loads were high in the nasal tissue with slightly higher bacterial titres in the nasal tissue of PCV13-vaccinated compared to unvaccinated mice (Figure 7B). While PCV-13 did not alter SPN loads in the BAL fluid of VEH-exposed animals, vaccination lead to a significant decrease in pneumococcal CFU in the HDM-exposed group compared to HDM-unvaccinated mice (Figure 7C). Immune cell infiltration in the BAL compartment was next assessed where differential cell counting revealed that macrophage/monocyte infiltration remained significantly higher in PCV13-vaccinated HDM-SPN mice (Figure 7D). A significant increase in the total number of neutrophils was observed in the PCV13-SPN group compared to VEH (Figure 7E); however, this did not differ to PCV13-HDM-SPN animals. In addition, HDM exposure alone also resulted in significant BAL eosinophilia in PCV13-vaccinated animals and numbers were significantly higher in vaccinated HDM-SPN animals compared to vaccinated HDM-exposed animals (Figure 7F). Hence, the BAL inflammatory profile in vaccinated mice is very similar to unvaccinated mice.

## Discussion

Our results confirm that animals with a HDM-driven allergic airway phenotype are inadequate at clearing pneumococci from the lower airways, as observed previously (Habibzay et al., 2012). To the best of our knowledge, our study describes for the first time how specific facets of macrophage and neutrophil anti-pneumococcal immunity are altered following aeroallergen exposure. We also show that prior PCV13 vaccination modestly accelerates pneumococcal clearance in HDM-exposed animals. Our study demonstrates that during the acute phase of infection, SPN lung titres are over 2-log higher in HDM-exposed mice despite the presence of nearly double the number of BAL macrophages/monocytes in the airways. AMs must engulf and internalise SPN (Franke-Ullmann et al., 1996) before using protease- and nitric-oxide dependant apoptotic pathways for bacterial removal (Marriott et al., 2004; Marriott et al., 2006). A similar phenomenon has been observed in AMs isolated from adults with severe asthma in response to *H. influenzae* and *S. aureus* (Liang et al., 2014). Consequently, a reduction in the phagocytic capacity could contribute towards bacterial outgrowth in the lower airways and may in part underpin why asthmatics have a heightened risk of developing pneumococcal pneumonia (Jounio et al., 2010; Castro-Rodriguez et al., 2020).

Pneumococcal infection in mice results in the robust recruitment of monocytes at day 2 post infection under the direction of the CCL2 chemokine, which enhances the killing of pneumococcus (Winter et al., 2007; Anthony et al., 2020). This recruitment is necessary to replenish local macrophage pools because SPN can induce apoptosis of resident macrophages to enhance pneumococcal killing (Aberdein et al., 2013). It has been reported that approximately 60% of alveolar macrophages are replaced by monocyte derived CD11b+ exudative macrophages following pneumococcal lung infection (Taut et al., 2008). The A limitation of our study is that we did not differentiate between resident alveolar macrophages, monocytes and monocyte derived exudative macrophages. As we have investigated the outcomes at Day 2 post infection, there will be a significant pool of monocyte derived macrophages in the lungs. Our findings suggest that this recruited pool is defective in bacterial clearance as pneumococcal load was 4-log higher in HDM-SPN infected mice compared to SPN-infected mice. Future studies should isolate the individual macrophage populations and investigate their relative capacity to ingest and kill SPN in order to establish whether this defect is shared or restricted across discrete macrophage subsets. In addition, female mice were used in this study as the prevalence of asthma is higher in females then males after puberty. Future studies should investigate whether defective pneumococcal clearance is also seen in male mice exposed to HDM aeroallergen.

Th2 immune cytokine, such as IL-4, IL-5, and IL-13 drive the emergence of AAMs. It was evident from our observations that HDM exposure generated a dominant AAM phenotype, as shown by the increased expression of typical AAM markers ARG1, YM1, IL-10, and FIZZ1 in the lung tissue and from isolated AMs. The polarisation of AAMs with IL-4 impairs the non-opsonic phagocytic uptake of *N. meningitidis*, which was associated with a defect in phagosome formation (Varin et al., 2010). We demonstrate that combined HDM exposure and SPN infection significantly increases the expression of ARG1 and IL-10 in murine lungs. IL-13-induced ARG1 expression in murine lungs has been shown to attenuate pneumococcal killing (Knippenberg et al., 2015). The upregulation of arginase depletes arginine and supresses iNOS protein expression, in turn decreasing NO production (El-Gayar et al., 2003). Since iNOS and NO production is important for reducing pneumococcal viability in macrophage-mediated phagocytosis, their suppression by increased arginase may result in loss of bacterial clearance. In addition, the bacterial virulence factor pneumolysin (PLY) increases the expression of arginase, which promotes endothelial barrier dysfunction by reducing nitric oxide generation (Lucas et al., 2012).

A fine balance between attenuating excessive inflammation without compromising bacterial clearance must exist. This especially applies to the role of IL-10 during anti-pneumococcal immune responses. Mice deficient of IL-10 display severe lung histopathology, sustained neutrophil infiltration and increased mortality following SPN infection [258]. Interestingly, pneumococcal titres in the lungs were significantly decreased in IL-10^−/−^ mice compared to WT (Peñaloza et al., 2015). Conversely, others have found that IL-10 diminishes pneumococcal clearance in the lungs and promotes bacterial spread (Peñaloza et al., 2015). Therefore, it is plausible that in our study increased IL-10 expression could contribute towards diminished SPN clearance in the airways. On the other hand, IL-10 plays an important role in reducing airway inflammation in OVA-induced asthma as IL-10 deficient mice display greater peribronchiolar inflammation consisting of lymphocytes, macrophages and eosinophils (Tournoy et al., 2000). Considering the multifaceted role IL-10 plays in infection and in allergy, a greater understanding of IL-10 during asthma exacerbations is needed.

We propose that mediators released in the allergic environment drive an alternately activated macrophage phenotype that is defective in pneumococcal clearance and killing. Whilst therapies such as corticosteroids can reduce allergic type-2 inflammation, they can also potentially suppress bacterial clearance. Hence, our pre-clinical model can be used to screen for novel therapies that improve bacterial clearance in the allergic lung environment. Another novel finding from our study is that HDM exposure attenuates the release of NETs in response to acute pneumococcal infection. Neutrophil-mediated removal of SPN involves both the killing of phagocytosed bacteria with stored serine proteases (El-Gayar et al., 2003) and the formation of NETs (Mori et al., 2012). As their name suggests, NETs form net-like structures that trap and kill pathogens (Brinkmann et al., 2004). In addition to neutrophils, eosinophils also produce extracellular traps. To highlight the presence of NETs, we co-stained lung sections with MPO and histone H3, and specifically measured the overlap of these two markers. There was also a significant reduction in dsDNA in the BAL fluid. The reduction in NETosis in HDM-SPN mice was not due to a decrease in the number of infiltrating neutrophils, as similar number of neutrophils were recruited to the airways in HDM-SPN and SPN groups. Key modulators of NETosis are still being defined. However, it has been highlighted that anti-inflammatory cytokines can inhibit the formation of NETs (Vorobjeva and Chernyak, 2020). Increased IL-10 production by dendritic cells disrupts the formation of NETs; a mechanism employed by HIV-1 to avoid NET-mediated clearance (Saitoh et al., 2012). Therefore, it is possible that increased IL-10 expression in HDM-exposed animals could limit NETosis in our model and enable SPN to evade NET-dependant anti-bacterial responses. SPN can also express a surface endonuclease allowing the bacterium to escape NETosis by breaking down DNA structures (Beiter et al., 2006). It is possible by virtue of the increased number of pneumococci in the lower airways of HDM-exposed mice (via impaired alveolar macrophage phagocytosis), that endonuclease expression is higher allowing for NET degradation.

The present study shows that acute SPN infection exacerbates airway hyperresponsiveness in HDM-exposed mice, which was associated with increased airway inflammation and mucus production. In addition, we showed that CCL2 expression in the lungs is significantly greater in HDM-SPN mice and is likely to drive the recruitment of monocytes to the airways in our model. While eotaxin-1 expression was not markedly higher in combined HDM-SPN animals, increased CCL3 expression may promote the recruitment of eosinophils in murine lungs. Interestingly, administration of the pneumococcal conjugate vaccine (PCV7) can supress type-2 inflammation in OVA-sensitised mice via increasing the induction of suppressive T-regulatory (Treg) cells (Thorburn et al., 2010). We investigated whether pneumococcal vaccination also improved bacterial clearance in HDM-exposed mice, as to the best of knowledge, this has not been described before. It was evident from our study that prior PCV13 vaccination modestly accelerated pneumococcal clearance in HDM-exposed mice, however as the reduction in bacterial load was small, BAL inflammation was still evident.

In this study, we specifically investigated the clearance of SPN during the early phase of infection (2 days post infection), where neutrophils and monocytes are recruited into the airways and play a major role in phagocytosing and killing pneumococcus. The significance of this early innate response is exemplified by the finding that CXCR2 knockout mice that recruit significantly fewer neutrophils and exudative macrophages during pneumococcal infection, display a major defect in bacterial clearance (Herbold et al., 2010). Our data suggests that this early innate response required to eradicate bacteria is defective in HDM-exposed mice. A limitation of our study is that we did not evaluate bacterial BAL titres at later timepoints following vaccination and pneumococcal infection (e.g., Day 3, 4, and 6), as it is plausible that vaccinated mice clear bacteria more effectively at the later timepoints. Furthermore, we did not evaluate antibody titres generated against PCV13 and/or pneumococcus. Future studies should investigate whether antibodies titres against PCV13 are altered in HDM-exposed mice and whether this impacts bacterial clearance at later timepoints. Nonetheless, our findings emphasise that there is a need to develop novel therapies that decrease type-2 and neutrophilic inflammation whilst improving phagocytosis and clearance of respiratory pathogens such as pneumococcus, and our pre-clinical model can be used to screen for such therapeutics.

In summary, we have shown that HDM-exposure reduces pneumococcal-specific AM-mediated phagocytosis and the formation of NETs; a phenomenon which may underpin the observed increase in SPN titres in HDM-exposed animals. We have also demonstrated PCV13-vaccination can enhance pneumococcal clearance in HDM-exposed mice. Our findings provide exciting new insights into the relationship between pneumococcal lung infections and allergic airways disease. The scope of this defect in asthma is accentuated by the fact that there is also a complex interplay between respiratory pathogens and asthma, where respiratory viruses such as rhinovirus can impair the phagocytic clearance of bacteria by human alveolar macrophages (Oliver et al., 2008). Future research should continue to focus on developing therapies that reduce bacterial loads and immunopathology in the background of allergic airways disease, and the pre-clinical model described in this study can contribute to progressing this strategy.

## Data Availability

The original contributions presented in the study are included in the article/Supplementary Material, further inquiries can be directed to the corresponding author.
